# A Relationship between Epithelial Maturation, Bronchopulmonary Dysplasia, and Chronic Obstructive Pulmonary Disease

**DOI:** 10.1155/2012/196194

**Published:** 2012-12-24

**Authors:** Abraham B. Roos, Tove Berg, Magnus Nord

**Affiliations:** ^1^Respiratory Medicine Unit, Department of Medicine, Karolinska Institutet and Lung Research Laboratory, L4:01, Karolinska University Hospital, Solna, 17176 Stockholm, Sweden; ^2^Department of Pathology and Molecular Medicine, McMaster Immunology Research Centre, McMaster University, Hamilton, ON, Canada L8S 4K1

## Abstract

Premature infants frequently develop bronchopulmonary dysplasia (BPD). Lung immaturity and impaired epithelial differentiation contribute together with invasive oxygen treatment to BPD onset and disease progression. Substantial evidence suggests that prematurity is associated with long term pulmonary consequences. Moreover, there is increasing concern that lung immaturity at birth may increase the risk of developing chronic obstructive pulmonary disease (COPD). The mechanisms contributing to this phenomenon remains unknown, largely as a consequence of inadequate experimental models and clinical follow-up studies. Recent evidence suggests that defective transcriptional regulation of epithelial differentiation and maturation may contribute to BPD pathogenesis as well as early onset of COPD. The transcriptional regulators CCAAT/enhancer-binding protein (C/EBP)**α** and C/EBP**β**, SMAD family member (Smad)3, GATA binding protein (GATA)6, and NK2 homeobox (NKX)2-1 are reported to be involved in processes contributing to pathogenesis of both BPD and COPD. Increased knowledge of the mechanisms contributing to early onset COPD among BPD survivors could translate into improved treatment strategies and reduced frequency of respiratory disorders among adult survivors of BPD. In this paper, we introduce critical transcriptional regulators in epithelial differentiation and summarize the current knowledge on the contribution of impaired epithelial maturation to the pathogenesis of inflammatory lung disorders.

## 1. Introduction

Premature infants with immature lungs frequently develop respiratory distress syndrome (RDS). Bronchopulmonary dysplasia (BPD) is the most common chronic respiratory disorder observed among premature infants with severely immature lungs [1, 2]. It is well established that BPD develops as a consequence of lung immaturity together with invasive critical care treatment regiments. The precise role of lung immaturity in this process is still relatively unknown, although several studies have tried to address this. Substantial evidence that airway dysfunction and long term morbidity can be observed among children and young adults surviving BPD has emerged [3–6]. In addition, there are increasing concerns that prematurely delivered individuals are more susceptible to chronic obstructive pulmonary disease (COPD) [4, 5, 7–10]. Experimental data support that defective transcriptional regulation of epithelial differentiation and maturation contributes to inflammatory processes involved in long term respiratory outcomes in immature lungs [11]. There is in light of this a pressing need to investigate long term consequences of prematurity in large clinical studies, and additionally address the contribution of epithelial immaturity at birth to this pathology. Increased knowledge of these mechanisms could translate into improved treatment strategies and reduced frequency of respiratory disorders among adult survivors of BPD.

## 2. Bronchopulmonary Dysplasia

The lungs of infants with very low birth weight (<1000 g) are severely immature, and pulmonary complications are common among these children. Specifically, respiratory distress syndrome (RDS) frequently develops [1], with BPD being the most common chronic respiratory disease seen with routine treatment of RDS [2]. BPD occurs in approximately 20% of all infants with a birth weight less than 1500 g [5] and develops as a consequence of elevated oxygen and ventilator-induced injury on the immature lung [12]. The predominant definition of BPD is based on gestational age together with the demand of oxygen supplementation. BPD is defined by the National Institutes of Health to occur among infants delivered before week 32 of gestation (postmenstrual age/midsaccular stage) who require supplemental oxygen treatment for at least 28 days after birth. The disorder is considered to be severe when the need for oxygen persists after week 36 [2]. The phenotype of the disease has, however, changed with new critical care regiments. These include less aggressive mechanical ventilation along with routinely administered exogenous surfactants and prenatal glucocorticoids. This has led to the classification of new BPD and old BPD. The histopathology of children born in the presurfactant era (old BPD) is dominated by a decreased alveolarization together with epithelial squamous metaplasia and epithelial hyperplasia, smooth muscle hyperplasia and remodeling of the pulmonary arteries, and fibroblast hyperproliferation with epithelial and mesenchymal remodeling. New BPD is in contrast less severe and is dominated by a disruption of distal lung maturation due to interruption of normal gestational growth, resulting in fewer and larger alveoli. BPD is presently observed in preterm infants delivered between 24 to 26 weeks of gestation, late in the canalicular period of lung development [13, 14]. The alveolar space enlargement and alveolar simplification in new BPD have been determined to be a result in an impairment in the processes of postnatal alveolarization [12], and is associated with modest airway epithelial remodeling and a varying degree of arterial remodeling, as well as fibroblast and smooth muscle cell proliferation [13, 14]. Yet, airway and alveolar remodeling still occurs. This highlights the need to increase the knowledge of the mechanisms that control epithelial differentiation and maturation.

## 3. The Airway Epithelium

The lung epithelium consists of the conducting epithelium lining the trachea, bronchus, and bronchioles, and the respiratory epithelium lining distal lung, including the alveoli. Several distinctly different epithelial cell types that can be grouped as basal, ciliated, secretory, or neuroendocrine cells constitute the conducting epithelium. Key processes that prepare the lung for the first breath, including epithelial cell differentiation and the initiation of surfactant production take place during late lung development [15, 16]. Thus, late stage lung development is vital for the viability of premature infants [16, 17]. In light of the critical role of lung immaturity in BDP pathology, a more profound knowledge of mechanisms that control lung maturation is key to a more profound understanding of this disorder. 

The trachea and main bronchi consist of a pseudostratified columnar epithelium, predominated by ciliated and basal cells, and interspersed with mucus cells. The more distal parts of the conducting airways consist of a simplified columnar epithelium with ciliated cells and Clara cells. Finally, the distal respiratory bronchioles consist of a simple cuboidal cell layer with only ciliated cells and Clara cells [18, 19]. The different functions of the specialized epithelial cells of the airways are critical for lung homeostasis, and disruption of this balance is observed in various lung disorders, including BPD and COPD. Ciliated cells are with unidirectional cilia beating critical for mucociliary clearance of foreign particles and microorganisms. Goblet cells, which secrete mucus that traps pathogens and particles, are also important in pulmonary host defenses [20]. In line with this, decreased cilia length is observed among smokers [21], and extensive ciliated cell damage appears to be present in BPD [22]. Furthermore, goblet cell metaplasia with mucus hypersecretion is a hallmark of COPD. This suggests that cigarette smoke changes the architecture of the airways, although the specific genetic factors that predispose certain individuals to these pathological changes yet remain to be determined. It is, however, possible that impaired differentiation programs during in utero development contribute to this pathology, although this has not been adequately addressed. Another secretory cell type in the airway epithelium, the Clara cell, produces host defense molecules such as secretoglobin/Clara cell secretory protein (SCGB1A1/CCSP/CC16) and the surfactant proteins (SP)-A and SP-D. Clara cells thus participate in pulmonary host defenses, and additionally serve to metabolize toxic substances through the production of P450 enzymes [23, 24]. Decreased number of Clara cells [25] and reduced expression of SCGB1A1 are observed in prematurely born infants that develop BPD [26, 27], as discussed in more detail below. The expression of SCGB1A1 is also decreased in patients with COPD [28].

## 4. The Alveolar Epithelium

**Figure 1:**
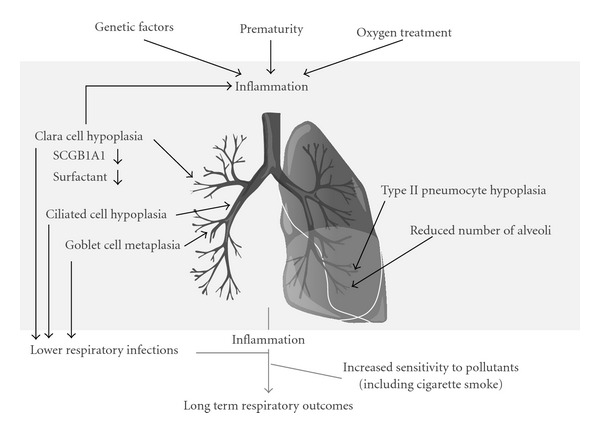
A proposed model for how airway epithelial maturation influences BPD and long term respiratory outcomes of prematurity. Prematurity, oxygen treatment, and genetic factors contribute to the pathology of bronchopulmonary dysplasia (BPD). Impaired epithelial maturation leads structural changes, disturbed epithelial homeostasis, and inflammatory responses and increased susceptibility to infections. Early life events such as lower respiratory infections and exposure to air born pollutants likely aggravate inflammatory processes initiated by prematurity.

Two highly specialized epithelial cells reside in the distal alveolar region of the lung, the flat and elongated type I pneumocyte and the cuboidal type II pneumocyte. Alveolar type I cells, derived from type II cells, facilitate gas exchange, and type II cells produce surfactant [29]. Surfactant lowers alveolar surface tension and has important functions in protecting the lung from pathogens [29, 30]. Thus, a fully mature and differentiated airway epithelium is not only critical for respiration but also provides a physical barrier with mucociliary trapping and removal of pathogens and foreign particles, and furthermore produces and secretes antimicrobial peptides, which are critical for pulmonary host defenses. Some studies suggest that airway branching is inhibited by macrophage derived inflammatory signaling dependent on nuclear factor (NF)*κ*B [31, 32], leading to a BPD-like phenotype in mice. It is, however, currently unknown how this inflammatory response is initiated, and what the cause is. It is well established that the pulmonary epithelium secretes chemokines that attract leukocytes to inflammatory foci, and cytokines that activate cells within the innate and adaptive immune response. Thus, it is possible that the pulmonary epithelium initiates the inflammation driving BPD pathogenesis (Figure 1). Furthermore, as it is currently unknown how impaired or defective epithelial differentiation alters inflammatory responses, future research efforts should be aimed at investigating whether different epithelial subtypes have specific roles in immune signaling.

## 5. Factors That Influence Lung Development and Contribute to BPD Pathology

### 5.1. C/EBP Transcription Factors

Many features of BPD, including the pulmonary inflammation associated with preterm labor, as well as the lung injury induced by hyperoxia and high pressure of mechanical ventilation, are recapitulated in lambs and baboons. Postnatal hyperoxia is also frequently used in mice and rats to produce a BPD-like phenotype [12]. Some transgenic mice are, however, born with developmental defects resembling BPD, without oxygen treatment. For instance, we and others have demonstrated that mice lacking the transcription factor CCAAT/enhancer-binding protein (C/EBP)*α* display enlarged airspaces with prominent interstitial tissue in the alveolar septa, when analyzed just before birth [11, 33, 34]. C/EBPs are ubiquitously expressed basic region-leucine zipper transcription factors [35, 36]. C/EBP*α*, C/EBP*β*, and C/EBP*γ* are enriched in the lung. C/EBP*α* was identified by McKnight and coworkers as a factor in hepatic nuclei capable of binding to the CCAAT box motif present in many different gene promoters [37]. C/EBP*β* was later identified as a protein that binds to an interleukin (IL)-1 responsive element in the gene coding for IL-6 [38]. Four additional members were subsequently identified and named according to the Greek alphabet (C/EBP*α*, *β*, *γ*, *δ*, *ε*, *ζ*) [35, 36]. The lung-enriched C/EBPs have been demonstrated to bind to virtually identical DNA sequences [39–43]. Functional replacement by different C/EBPs is thus conceivable, as has been suggested previously [44]. C/EBP*α* has pleiotropic functions [43] but is particularly important for terminal cell differentiation of the lung epithelium. Mice deficient for C/EBP*α* exhibit impaired differentiation of the pulmonary epithelium, especially in the alveolar region, with airspaces lined with immature cuboidal cells in place of type I pneumocytes. The production of surfactant proteins is also impaired in these mice [11, 33, 34]. The alveolar phenotype of pre- or postnatal mice lacking C/EBP*α* is consistent with the pathological lesions observed in preterm infants with pulmonary immaturity, including decreased surfactant production [13, 14]. It is, however, important to point out that these changes are due to a defective developmental program, and not inflammatory responses caused by invasive oxygen treatment, which is suggested to dominate progression of BPD pathology. Nevertheless, others have demonstrated a key role for C/EBP*α* in the pathological changes caused by hyperoxia. Xu and colleagues used inducible Cre to delete C/EBP*α* specifically in the lung epithelium after birth. While both differentiation of the cells lining airways and alveolar spaces, as well as alveolar septation were retained, with normal lung structure, an amplified inflammatory response to hyperoxia causing severe damage is observed in these mice, suggesting that C/EBP*α* is required for cytoprotection [45]. Another study using siRNA to target *Cebpa *found that mice with inhibited expression of C/EBP*α* display hyperproliferation and impaired type II pneumocyte differentiation after recovery from hyperoxic challenge [46]. In addition, a recent study documented a critical role for C/EBP*α* in regulating the repair processes via regulation of the protease balance following airway damage [47], suggesting that C/EBP*α* is key in protecting the pulmonary epithelium. These findings support a central role for C/EBP*α* and pulmonary epithelial maturation in protecting against pathological changes associated with BPD. Altogether, these findings highlight that maturation level at time of birth is crucial in the protection against the damaging effects of hyperoxia treatment. 

### 5.2. Impaired Airway Epithelial Differentiation

Extensive research has focused on oxygen-induced damages but as BPD remains common with more sophisticated clinical practices, the role of genetic components should not be overlooked. Genetic variations are likely to contribute to BPD, as well as the long term respiratory outcomes of prematurity. Several studies implicate the Clara cell derived protein SCGB1A1 in respiratory disorders. As mentioned earlier, the expression of SCGB1A1 is reduced in prematurely born infants that develop BPD [26, 27], as well as in patients with COPD [28]. In premature infants with (new) BPD, the reduced expression is associated with a decreased number of Clara cells in the bronchiolar epithelium [25]. Thus, Clara cells could potentially play important roles in these pulmonary disorders, although it still remains to be addressed whether the reduced number of Clara cells in premature infants contributes to the development of COPD. Studies investigating this could provide important insights and increase our currently incomplete knowledge of the processes that influence airway pathology. In addition to a central role in host defenses, Clara cells are suggested to serve as stem cells for the respiratory epithelium and give rise ciliated cells and mucus cells. In inflammatory conditions, Clara cell transdifferentiation into a mucus cell linage represents a possible mechanistic explanation to goblet cell hyperplasia. The current data does not confirm the presence of goblet cell hyperplasia in BPD [22], although the available documentation is incomplete due to the difficulty in obtaining clinical samples. Clara cell hypoplasia could indicate diminished host defenses, as well as a deficient stem cell pool with possible long term consequences on normal epithelial turn over and regeneration. How Clara cell transdifferentiation to goblet cells is mechanistically controlled is not completely understood but several transcription factors and signaling molecules have been identified as regulators and mediators of this differentiation pathway. The SAM pointed domain-containing ETS transcription factor (SPDEF) is implicated in this process, as well as transcription factors involved in goblet cell differentiation such as forkhead box (Fox)A3, whereas postnatal Notch signaling prevent differentiation into mucus cells [48]. C/EBP*α* and C/EBP*β* deficiencies also lead to goblet cell metaplasia, together with a complete absence of Clara cells, supporting a role for C/EBP transcription factors in inhibiting goblet cell differentiation in support of the Clara cell linage [44]. Taken together, Clara cell hypoplasia may reduce the expression of host defense molecules, impair epithelial regeneration, and thus contribute to BPD pathogenesis. Targeting the underlying mechanisms that regulate Clara cell transdifferentiation to goblet cells, such as stimulating the expression of C/EBP transcription factors, may prove to be valuable in preventing long term respiratory pathogenesis in preterm infants. Furthermore, stimulation of C/EBP*α* signaling could prove beneficial to increase alveolar maturation in prematurely delivered children. 

### 5.3. Glucocorticoids

Transgenic mice with defects in glucocorticoid signaling are also born with impaired lung development resembling BPD. Glucocorticoids have been established as vital for maturation of the lung epithelium, specifically for differentiation of the type II cells of the alveolar region and onset of surfactant production [49–53]. In addition, rats lacking the glucocorticoid receptor specifically in the lung epithelium express lower levels of SCGB1A1 [54]. In line with a critical role in lung development, glucocorticoid therapy is central in increasing the survival rate for prematurely delivered infants, although adverse effects of glucocorticoids on neurodevelopment must be carefully considered before treatment is initiated. Although it is well established that the glucocorticoid receptor acts as a transcriptional activator, the mechanism involved in epithelial maturation appears to be independent of transactivation. Evidence suggests that the glucocorticoid-induced maturation of the pulmonary epithelium is in part mediated through activation of C/EBP transcription factors [55–57]. For instance, glucocorticoid-induced transactivation of *Scgb1a1* occurs through activation of C/EBP*β* and C/EBP*δ* [57]. Also supporting a link between glucocorticoids and C/EBPs, mice lacking C/EBP*α* recapitulate the developmental phenotype of mice lacking the glucocorticoid receptor [58]. Furthermore, glucocorticoid therapy represents an attractive method of stimulating C/EBP signaling [59], which may be beneficial in BPD therapy (discussed above). On the other hand, it has been demonstrated that glucocorticoids impair late stage alveologenesis, in a comparable fashion to mice overexpressing C/EBP*α* [60] with decreased alveolar septation and defective vascularization [61]. This indicates that glucocorticoids should be administered in a strict temporal fashion to prevent alveolar simplification [62]. Additional studies investigating the underlying mechanisms of the beneficial effects of glucocorticoids on alveolar cell maturation and surfactant production, versus the potential negative effects, are imperative to increase the current knowledge. In addition, the possible benefit of continuous glucocorticoid treatment in children surviving BPD should be evaluated, as glucocorticoids could be important in suppressing the inflammatory processes that may contribute to long term respiratory outcomes. Glucocorticoids may through activation of C/EBPs have beneficial effects in reducing the incidence of obstructive airway disease in children surviving BPD in part by inhibiting goblet cell hyperplasia. A similar effect in reducing goblet cell hyperplasia is observed in COPD patients treated with budesonide [62], although the involvement of C/EBPs has not been addressed in this context.

## 6. A Link between Prematurity and Chronic Obstructive Airway Disease

### 6.1. An Increased Risk of Asthma among Premature Children

A substantial amount of evidence supports that the lung injury associated with BPD translates into an increased risk of developing asthma. Asthma is reported to be twice as common among premature children, as compared to children born at full term [4]. Another, more recent study supports that either short gestational duration or low birth weight alone increases the risk of developing asthma [6]. Available data from animal models support that hyperoxia causes airway remodeling and hyperreactivity. Experimental studies also suggest that this hyperreactivity is associated with inflammatory cell infiltrates [63, 64]. There is also increasing concern that the premature children are more susceptible to pulmonary insults such as infections or cigarette smoke exposure, which may lead to chronic conditions and obstructive airways in adulthood. This predisposition may be related to a continued inflammatory process in the pulmonary tissue. One study found higher levels of a series of inflammatory mediators, including interferon (IFN)-*γ*, monocyte chemotactic protein (MCP)-1, IL-6, IL-13, and IL-17 in premature children one year after birth, suggesting a persistent inflammatory process [65]. Supporting that this sustained inflammation has a pathological relevance, abnormal baseline spirometry, and impaired exercise capacity and significantly more respiratory symptoms are noted among childhood survivors of extreme preterm birth. Furthermore, radiologic abnormalities have been observed in children and young adults born prematurely [3]. However, is currently not known how these lesions develop over time, as cumulative data has not yet been generated from the cohort with new BPD. There is, however, some evidence to suggest that adult survivors of BPD suffer from respiratory symptoms such as shortness of breath and wheeze, as well as airway obstruction [5]. Given that normal lung function decline with age, long term follow-up studies of children and adults surviving BPD will be important to identify potential increase in age-related decline in lung function. In these clinical studies, efforts should be made to investigate inflammatory cell infiltrates and inflammatory markers such as cytokines and/or exhaled nitric oxide. 

### 6.2. Increased Prevalence of COPD among Premature Children

It has been proposed that low birth weight can predispose individuals to develop COPD later in life [66]. COPD is characterized by progressive airflow limitation in response to noxious particles or gases. A dramatic increase in disease prevalence is predicted in the immediate future, with a considerable impact on global health [67]. COPD is diagnosed based on lung function, when the ratio between forced expiratory volume in one second (FEV_1_) and forced vital capacity (FVC) is <0.7 [68, 69]. A series of pathological lesions, including obstructive bronchiolitis, bronchitis, and emphysema contribute to airflow limitation in varying degree in different individuals [67]. In line with this, several different clinical phenotypes have been proposed to exist, which relate to meaningful outcomes [70]. Increased documentation of these clinical phenotypes is vital to develop novel therapeutic interventions, as well as to improve COPD management. In spite of substantial research efforts, it still remains uncertain exactly how COPD pathology develops and progresses [71]. It is, however, well established that cigarette smoke induces an inflammatory response that involves accumulation of neutrophils, macrophages, T lymphocytes, and B cells [72, 73], which causes destruction of lung parenchyma and airway remodeling that contribute to the airflow limitation [67]. Although cigarette smoking is the etiological factor in a vast majority of cases, only a minority of all smokers are estimated to develop airway obstruction [67]. This highlights the involvement of genetic components, possibly affected by developmental arrests and insults early in life, such as BPD. The natural course of lung function from birth to adulthood provides a simple explanation to how impaired lung function at birth may contribute to obstructive airwaysinadult life. Lung function (FEV_1_ and FVC) increases from childhood until early adulthood, whereafter it slowly declines. Once middle age is reached, the decline precedes more rapidly [74]. Abnormal lung function at birth decreases the peak lung function and may even accelerate the decline. Airway inflammation initiated by preterm delivery in combination with changes in the architecture of the lung, that is mucus cell hyperplasia and alveolar simplification, may also be contributing factors which predispose an individual to develop airway obstruction, even without cigarette smoke exposure. Data from animal models also support a link between BPD and COPD. Hyperoxia treatment of newborn pups increases sensitivity to cigarette smoke in adult mice, with increased emphysema and decreased surfactant production compared to control animals. The number of inflammatory cells is, however, not increased compared to mice exposed to room air [75], suggesting a central role of the hyperoxia-induced airspace enlargement, and possibly an increased vulnerability of the structural cells in hyperoxia treated mice. As it is furthermore proposed that COPD is a collection of different clinical conditions with nonreversible airway obstruction as a common hallmark, preterm infants with BPD may develop airway obstruction that is different from cigarette smoke-induced COPD. Large and well controlled studies of the clinical phenotypes are thus imperative. Further understanding of the processes that drive airway obstruction in different individuals is particularly important considering the increasing survival rates of prematurely born infants. 

## 7. The Possible Consequences of Impaired Transcriptional Regulation of Epithelial Maturation on Long Term Respiratory Health: A Link between BPD and COPD

### 7.1. C/EBP*α* in COPD Pathogenesis

One of the most striking findings in mice lacking C/EBP*α* specifically in the lung epithelium is that the pulmonary phenotype persists and is aggravated over time. A majority of the histopathological lesions observed in COPD are observed in adult mice lacking C/EBP*α*, including emphysema, bronchiolar ectasia, mucus plugging, and centrilobular interstitial fibrosis. These lesions develop spontaneously, without tobacco smoke exposure. This supports that genetic factors and early life events (i.e., pulmonary immaturity at birth) increase the risk of developing chronic lung disorders [4, 5, 7–10]. The continuous enlargement and/or destruction of alveoli may be mechanistically explained by a leukocyte accumulation [11] similar to what is observed in COPD [76, 77]. As there is no pulmonary phenotype when C/EBP*α* is deleted after birth [45], the lung immaturity, not the C/EBP*α* deficiency in itself, instigates the impaired lung homeostasis in adult life. It is thus possible that lung immaturity and impaired epithelial differentiation/maturation could play a key role in initiating the inflammatory processes that drive the progression of emphysema regardless of the invasive oxygen treatment. 

### 7.2. C/EBP*β* in COPD Pathogenesis

While mice lacking C/EBP*β* specifically in the lung epithelium are not reported to have any developmental impairment, this factor contributes to gene regulation together with C/EBP*α*. Mice lacking both factors display a more severe pulmonary phenotype, suggesting a compensatory mechanism [44]. Furthermore, the activity of C/EBP*β* is increased in smokers [78], and C/EBP*β* is critical for the inflammatory response to lipopolysaccharide [79] as well as cigarette smoke [78] in the airway epithelium. As C/EBPs also are critical for expression of host defense molecules such as surfactants and SCGB1A1 [44], this suggests that these factors play a role in pathogen resistance. Severe childhood wheeze is twice as common among preterm infants who have experienced a lower respiratory tract infection, as compared to preterm children without a reported infectious incident [80], suggesting that infections contribute to respiratory pathology in preterm infants. A recent meta-analysis concluded that childhood pneumonia also affects long term respiratory outcomes, with an increased risk for restrictive lung disease, obstructive lung disease, and bronchitis among children that had experienced a severe infection [81]. Although the mechanisms underlying this phenomenon have not been addressed, reduced pulmonary surface area, hyperoxia-induced damages of the endothelial surfaces, and impaired cellular immunity among preterm infants may serve to explain the increased susceptibility to infections [82]. Reduced expression of host defense molecules is also likely to contribute to the increased prevalence of respiratory infections among preterm infants with or without BPD, and airway remodeling undoubtedly influences the pathogenesis of disorders such as bronchitis. Thus, C/EBPs may influence long term respiratory outcomes by regulating host defenses to respiratory infections and are likely to play intricate roles in lung maturation and pathogenesis of smoking-related lung diseases. In conclusion, C/EBPs are possible factors that link lung immaturity to long term respiratory outcomes. 

### 7.3. Other Critical Transcriptional Regulators

Other intervention studies have shed light on key processes in the regulation of lung maturation and epithelial differentiation, as well as the possible consequences on pulmonary disease in adult life. These studies have also advanced our knowledge of mechanisms involved in the pathogenesis of BPD. SMAD family member (Smad)3/transforming growth factor (TGF)-*β* signaling has emerged as a potentially important target for therapeutic intervention, as mice with disrupted Smad3/TGF-*β* signaling show similarities with the BPD phenotype [83] and display aggravated emphysema in response to cigarette smoke exposure [84]. Several other transcriptional regulators are also identified as important for the formation of alveolar spaces and epithelial differentiation, and may be involved in pathological changes associated with COPD. These include the transcription factors GATA binding protein (GATA)-6 and NK2 homeobox (NKX)2-1 [85, 86]. NKX2-1 has been demonstrated to synergistically regulate the *Scgb1a1* gene together with C/EBP*α* [87]. Other studies have shown that NKX2-1, which is imperative for normal lung formation [88], represses expression of the *Muc5b* mucin gene, inhibits mucus cell metaplasia, and favors a T helper (Th)2 immune response. On the other hand, overexpression of NKX2-1 in mice causes type II pneumocyte hyperplasia and emphysema associated with severe pulmonary inflammation and fibrosis [89]. This is similar to the phenotype in mice overexpressing or lacking C/EBP*α* [11, 60]. GATA-6, in contrast to NKX2-1 transcriptionally activates *Muc5b* and appears to promote goblet cell differentiation [90, 91]. The winged helix transcription factor FOXA2 is also established as central in epithelial differentiation, as well as inflammatory responses. Loss of FOXA2 induces a Th2 cell-mediated response, expression of SPDEF, and differentiation towards the goblet cell linage. In addition, mice lacking FOXA2 specifically in the lung epithelium exhibit enlarged airspaces and neutrophil accumulation in the respiratory tract in similarity to mice overexpressing NKX2-1, as well as goblet cell hyperplasia with increased mucin production [92, 93]. These findings support that transcription factors important for epithelial differentiation and lung maturation also may play a role in the pathology linked to COPD. Thus, premature delivery with halted lung maturation could affect inflammatory processes that contribute to the pathogenesis of airway obstruction later in life. 

## 8. Conclusions

Mechanistic studies investigating the transcriptional regulation of lung development and epithelial differentiation are important not only to understand respiratory failure in premature infants but also to enhance our knowledge of the processes that contribute to pulmonary diseases in adult life. Several transcription factors that control lung organogenesis, branching morphogenesis, and epithelial differentiation and maturation have been implicated in pulmonary disorders among adults. A more comprehensive understanding of developmental processes in the lung may thus be important to identify potential targets for medical interventions in patients with inflammatory pulmonary diseases. Further studies are, however, needed to determine to what extent lung immaturity at birth predisposes individuals to pulmonary disorders.
